# The Influence of General and Local Muscle Fatigue on Kinematics and Plantar Pressure Distribution during Running: A Systematic Review and Meta-Analysis

**DOI:** 10.3390/sports11120241

**Published:** 2023-12-06

**Authors:** Walaaeldin Aly Hazzaa, Laura Hottenrott, Manar Ahmed Kamal, Klaus Mattes

**Affiliations:** 1 Department of Movement Science, Hamburg University, 20148 Hamburg, Germany; klaus.mattes@uni-hamburg.de; 2 Faculty of Physical Education for Boys, Training Science, Helwan University, Giza 11795, Egypt; 3 Institute of Performance Diagnostics and Health Promotion, Martin-Luther-University Halle-Wittenberg, 06108 Halle, Germany; hottenrott.laura@gmail.com; 4 Faculty of Medicine, Benha University, Benha 13518, Egypt; manar170393@fmed.bu.edu.eg

**Keywords:** running, local muscle fatigue, treadmill, plantar pressure distribution

## Abstract

Fatigue has the potential to alter how impact forces are absorbed during running, heightening the risk of injury. Conflicting findings exist regarding alterations in both kinematics and plantar pressure. Thus, this systematic review and subsequent meta-analysis were conducted to investigate the impact of general and localized muscle fatigue on kinematics and plantar pressure distribution during running. Initial searches were executed on 30 November 2021 and updated on 29 April 2023, encompassing PubMed, The Cochrane Library, SPORTDiscus, and Web of Science without imposing any restrictions on publication dates or employing additional filters. Our PECOS criteria included cross-sectional studies on healthy adults during their treadmill running to mainly evaluate local muscle fatigue, plantar pressure distribution, biomechanics of running (kinematics, kinetics, and EMG results), and temporospatial parameters. The literature search identified 6626 records, with 4626 studies removed for titles and abstract screening. Two hundred and one articles were selected for full-text screening, and 20 studies were included in qualitative data synthesis. The pooled analysis showed a non-significant decrease in maximum pressure under the right forefoot’s metatarsus, which was more than the left rearfoot after local muscle fatigue at a velocity of 15 km/h (*p*-values = 0.48 and 0.62). The results were homogeneous and showed that local muscle fatigue did not significantly affect the right forefoot’s stride frequency and length (*p*-values = 0.75 and 0.38). Strength training for the foot muscles, mainly focusing on the dorsiflexors, is recommended to prevent running-related injuries. Utilizing a standardized knee and ankle joint muscle fatigue assessment protocol is advised. Future experiments should focus on various shoes for running and varying foot strike patterns for injury prevention.

## 1. Introduction

During running, the foot is subjected to forces equivalent to two to three times the runner’s body weight. The lower extremity muscles are crucial in providing effective shock absorption to prevent incorrect or excessive loading of the passive musculoskeletal system, primarily through eccentric contraction of the plantar flexors [1]. When these muscles experience fatigue, their ability to adequately absorb impact forces diminishes, potentially affecting the passive musculoskeletal system and elevating the risk of running-related injuries. Over 90% of running-related issues are related to the lower extremities, with approximately one-third involving the knee, lower leg, and foot [2,3]. The annual incidence of lower extremity problems among runners ranges from 19.4% to 79.3% [4]. Numerous studies have demonstrated the correlation between muscular fatigue and the risk of injury [5,6]. However, despite this body of research, how fatigue specifically alters foot loading remains unclear. Understanding these changes in foot loading due to fatigue is vital for tailoring injury-preventative training recommendations, particularly for runners with a history of lower extremity injuries, who face an increased risk of re-injury [5,6]. In an ideal scenario, preventing running injuries before they manifest is crucial [3]. However, the surge in runners has been accompanied by a rise in associated health issues, predominantly affecting the lower limbs [7]. Multiple studies have highlighted the connection between fatigue and injury [3,8], yet the exact relationship between injuries and foot strike patterns remains elusive [9].

The exploration of the effects of general and muscle fatigue on plantar pressure distribution and increased ground reaction force during running has seen a significant increase in research since 2006. However, due to a wide array of results, the precise impact of fatigue remains unclear. In a meta-analysis examining the alteration of ground reaction force following fatigue, Zadpoor and Nikooyan [10] highlighted that most studies have primarily focused on the active peak vertical ground reaction force, given its reflection of muscular reaction during ground contact. Two major theories dominate the ongoing debate on this subject. The first theory posits that fatigue diminishes the capacity for adequate shock absorption, causing an increase in ground reaction force to counterbalance this effect.

On the other hand, the second theory suggests a reduction in ground reaction force due to the human body’s protective strategy to prevent injuries [10]. The meta-analysis incorporated eight studies that assessed the active peak of the ground reaction force using force plates before and after fatigue induced by running. Among these, three studies demonstrated a significant decrease [11,12,13], while four articles indicated minor, non-significant changes [14,15,16]. Another study explored ground reaction force during running after inducing local muscle fatigue in the dorsiflexors and invertors of the foot [17]. The findings revealed a non-significant increase in dorsiflexors and a reduction in ground reaction force due to inversion.

Other studies examining ground reaction forces have revealed conflicting results. Morin et al. [18] and Morin et al. [19] observed a significant reduction in ground reaction force after extreme fatigue induced by either a 24-h treadmill run or an ultra-mountain marathon. The decrease ranged from 2.24 to 2.14 times the body mass in one study and 2.32 to 2.17 times in another [18,19].

Quammen et al. [20] compared two different fatigue protocols. One protocol involved strength tests and sprints, while the other incorporated strength tests followed by a 30-min treadmill run. Surprisingly, both protocols led to a non-significant increase in the active peak of the ground reaction force.

Studies demonstrate varied plantar pressure distribution across different foot areas following running, ranging from 10 km to a full marathon and even 30-min runs [21,22]. Forefoot pressure specifically increases after running distances of 10 km to a marathon and during 30-min runs [21,22,23]; this heightened forefoot pressure is also noted after inducing local fatigue in the plantar flexors and dorsiflexors [24]. However, the effect of running-induced fatigue on pressure distribution across different foot areas remains unclear. Some studies have reported decreased pressure under the heel [2,21,22,25], while others have observed a significant increase [22,26]. Pressure under the toes was significantly reduced in some instances [2,24], while two other studies found no significant differences [21,26]. The lack of consensus can be attributed to variations in fatigue protocols and the specific foot areas examined. Another challenge lies in understanding how fatigue influences foot strike patterns during running. Although it is commonly assumed that fatigue alters foot strike patterns, conclusive evidence is lacking [27,28]. Fatigue may lead to runners modifying their foot strikes during running, potentially increasing the risk of injury [5,6]. Therefore, determining whether fatigue induces muscular imbalances and alters foot strike patterns is crucial for injury prevention [29].

Fatigue exerts an impact on both stride frequency and stride length during running. Some studies have shown a decrease in stride frequency and an increase in stride length [2,15]. However, there were also instances of only minor changes [30] or even no changes in stride frequency [25]. Additionally, treadmill running introduces alterations in stride frequency [2,31,32,33]. At moderate speeds on a treadmill, there is a tendency for a reduction in stride length and an increase in stride frequency compared to natural ground running [34]. However, it is essential to note that the influence of the treadmill on running movements has led to controversial discussions regarding the results [2]. Despite the controversies, these treadmill-induced changes have been accepted by various authors as representative of running investigations [31,32,35].

Regarding local muscle fatigue, the influence of muscle fatigue on the plantar flexors and dorsiflexors has been investigated in various publications. However, there is significant variability in methodologies, leading to varying muscle activation outcomes. A common limitation is the failure to consider critical factors like foot strike patterns, leg dominance, running speeds, distances, and repeated measurements. Many studies utilize exhaustive running, typically a 30-min run at 85% of the individual’s maximal aerobic speed, to induce fatigue [2,10]. However, distinguishing the changes directly resulting from local muscle fatigue remains challenging. The dorsiflexors and plantar flexors of the foot are highly susceptible to severe fatigue during running, considering their involvement in 50 to 85% of the running cycle [36]. Prior research has indicated that plantar flexors experience fatigue after a 2-h running session [37,38].

Similarly, running activities induce significant fatigue in the dorsiflexors [39,40] despite their primary activity during the swing phase. Notably, the muscular imbalance becomes evident during running with progressive fatigue. The activity of plantar flexors remains relatively constant, while that of dorsiflexors decreases, resulting in an imbalance [41].

In the domain of kinematics, studies have examined the ankle and knee angles of runners post-fatigue. Kellis and Liassou [36] highlighted that the knee and ankle angles during touch-down play a vital role in joint stability and are particularly crucial during toe-off. Bruggemann et al. [42] observed increased rearfoot angle during touch-down and delayed attainment of its maximum value after fatigue. Furthermore, Christina et al. [17] observed a decrease in the ankle angle following local muscle fatigue of the dorsiflexors, while fatigue of the plantar flexors contributed to a notable increase in the ankle angle. Fatigue of the ankle musculature due to movement resulted in a decrease in the dorsiflexor angle during the stance phase [36]. A reduced dorsiflexor angle signifies that a greater portion of the heel has contact with the ground during stance, facilitating enhanced absorption of landing forces [2]. This kinematic adaptation involves an increased knee flexor angle [43] and reduced ankle angle during the stance phase [17].

Therefore, the present review and meta-analysis aimed to examine the influence of general and local muscle fatigue on kinematics and plantar pressure distribution during running. It was assumed that the plantar pressure distribution under the foot would differ according to fatigue protocols; they submitted different results, and this is due to using other fatigue protocols.

## 2. Materials and Methods

### 2.1. Study Design and Protocol Registration

The present review strictly adhered to the preferred reporting items for systematic reviews and meta-analyses (PRISMA) guidelines. To maintain accuracy and rigor, diligent searches were conducted to identify any errata, corrections, corrigenda, or retractions related to the included studies [44]. Additionally, pre-registered protocols were retrieved when available to uphold transparency and reliability in the review process. To ensure a comprehensive overview, if a study provided supplementary and pertinent information in another published article, this data was integrated to enhance the completeness of the information. Moreover, the review protocol was registered in the University of York’s Centre for Reviews and Dissemination PROSPERO database under the registration number CRD42020202711, accessed on 19 September 2020. (http://www.crd.york.ac.uk/prospero/).

### 2.2. Inclusion and Exclusion Criteria

Inclusion and exclusion criteria were established following the participants, exposure, comparator, outcome, and study design (PECOS) framework. Inclusion criteria encompassed cross-sectional studies published in peer-reviewed journals focused on fatigue and plantar pressure distribution during treadmill running. Studies that delved into plantar pressure distribution, running biomechanics (including kinematics, kinetics, and EMG outcomes), and temporospatial parameters involving healthy adult runners were considered. On the other hand, exclusion criteria comprised studies reporting on individuals with preexisting medical pathologies such as diabetes, neuromuscular, or cardiovascular diseases. A comprehensive overview of the eligibility criteria can be found in (Table 1).

### 2.3. Search Strategy

Initial searches were conducted in PubMed (1950 to 30 November 2021, and updated on 29 April 2023), The Cochrane Library (1991 to 30 November 2021, and updated on 29 April 2023), SPORTDiscus (1977 to 30 November 2021, and updated on 29 April 2023), and Web of Science (Thomson Reuters, New York, NY, USA) (1945 to 30 November 2021, and updated on 29 April 2023), without restrictions on publication date and no filters applied. Subject headings, synonyms, relevant terms, and variant spellings for the searches on each database were used. Manual searches were conducted by screening the included studies and relevant review reference lists. The general search strategy used the following free terms without filters or limits applied: The complete eligibility criteria are listed in Table 1. We used the following keywords: (“run” OR “running” OR “jump”) AND (“fatigue” OR “exhaust” OR “tired” OR “exert” OR “prolong”) AND (“biomechanics” OR “kinematic” OR “kinetic” OR “ground reaction force” OR “electromyography” OR “plantar pressure”). Primarily, the databases were searched to prepare a list of functional studies based on the article title and abstract. Prospective snowballing citation tracking was performed in Web of Science on 17 December 2021 and updated on 29 April 2023.

### 2.4. Study Selection

Two independent reviewers (W.H. and M.A.) conducted the initial identification of relevant studies, with a third reviewer (K.M.) available for consensus in cases of disagreement. The identified studies were initially screened against the inclusion criteria, beginning with a title evaluation, then an abstract assessment, and finally, a thorough review of the full text. Furthermore, the bibliographical information of the included articles was meticulously examined to identify additional relevant references. Citation tracking was executed using the Web of Science (Thomson Reuters). For inclusion in this review, articles had to meet specific criteria, including cross-sectional studies published in peer-reviewed journals written in English or German. Conversely, reviews, systematic reviews, commentaries, case studies, and case series were excluded from the review process.

### 2.5. Data Extraction

Two independent reviewers (W.H. and M.A.) conducted the data collection, utilizing two separate Excel sheets: (1) baseline summary comprising study ID, title, country, study design, sample size, inclusion criteria, aim, primary outcome, secondary outcome, and conclusions; and (2) specific biomechanical data encompassing stride frequency, stride length, maximum pressure under the heel, maximum pressure under the metatarsus, and maximum pressure under the forefoot for both the right and left forefoot and rearfoot after local muscle fatigue. Notably, the data presented in this review primarily pertains to a running velocity of 15 km/h, as no significant differences were observed in the results for other velocities. In cases where data was missing, the authors of the included studies were promptly contacted to request the necessary information.

### 2.6. Quality Assessment

The risk of bias within the included studies was independently assessed by two reviewers (WH and MA) using the Joanna Briggs Institute (JBI) critical appraisal checklist for cross-sectional studies [45]. Each question in the checklist was evaluated as either “Yes,” indicating a low risk of bias, “No”, indicating a high risk of bias, “Unclear”, or “Not applicable”. To enhance the transparency and clarity of the risk-of-bias assessment, the Risk-of-bias Visualization (robvis) software was employed to visualize the appraisal outcomes [46].

### 2.7. Statistical Analysis

The statistical analysis used Review Manager (RevMan) software version 5.4. Results with a *p*-value (P) less than 0.05 were considered statistically significant in the Z-test. The meta-analysis results for continuous outcomes were presented using mean difference (MD) and a 95% confidence interval (CI). For the analysis, the fixed-effect model was utilized. To assess heterogeneity among the studies, the Chi-square test was employed to measure its significance. Potentially significant heterogeneity was defined if the *p*-value was less than 0.1. Furthermore, the degree of heterogeneity was evaluated using the I^2^ test [47]. The I^2^ statistic ranges from 0% to 100%, indicating low heterogeneity (25%), medium heterogeneity (50%), and high heterogeneity (75% or greater) [48]. This statistic gauges the extent of inconsistency across studies in a meta-analysis, providing valuable insights into result consistency. Notably, the I^2^ statistic can be directly compared between meta-analyses involving different numbers of studies and various types of outcome data. It is considered preferable to a test for heterogeneity in assessing the consistency of evidence [49].

## 3. Results

**Figure 1:**
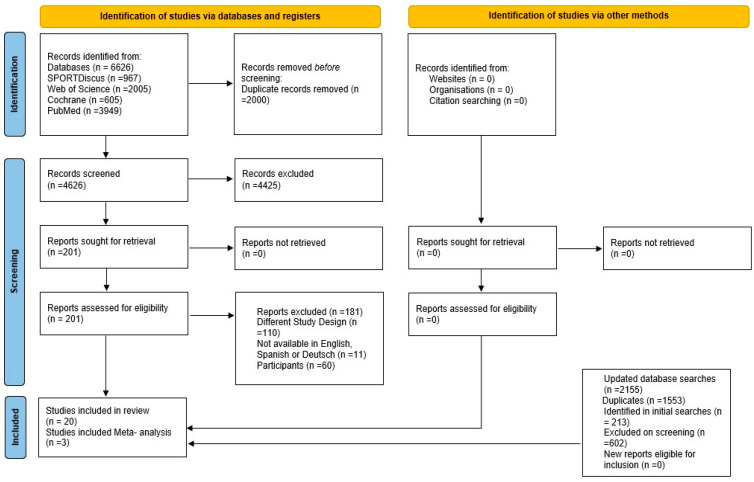
PRISMA (preferred reporting items for systematic reviews and meta-analyses) 2020 flow diagram.

The literature search yielded a total of 6626 records. After removing 2000 duplications through automated processes using EndNote™ 20.2 for Mac (Clarivate™) and subsequent manual screening, 4626 studies remained for the title and abstract screening. Following the title and abstract screening, 201 articles were selected for full-text screening. Eventually, 20 studies met the criteria for inclusion in our qualitative data synthesis, and among them, three studies were included in our meta-analysis (Figure 1).

### 3.1. Baseline and Summary of Included Studies

The studies were published between 1997 and 2020. Studies were performed mostly in Europe (Spain, France, Germany, the Netherlands, Switzerland, and the United Kingdom) (55%), [2,21,50,51,52,53,54,55,56,57,58] followed by North America (USA) (7%) [22,43,59,60,61,62,63], South America (5%) [62], and Asia (5%) [64], and no study was performed in Africa (0%). Most of the included studies were published by authors affiliated with the USA. The types of studies were cross-sectional studies with wide-ranged sample sizes (9–52). There were 431 athletes in the 20 studies included (Table 2).

### 3.2. Risk of Bias

**Figure 2:**
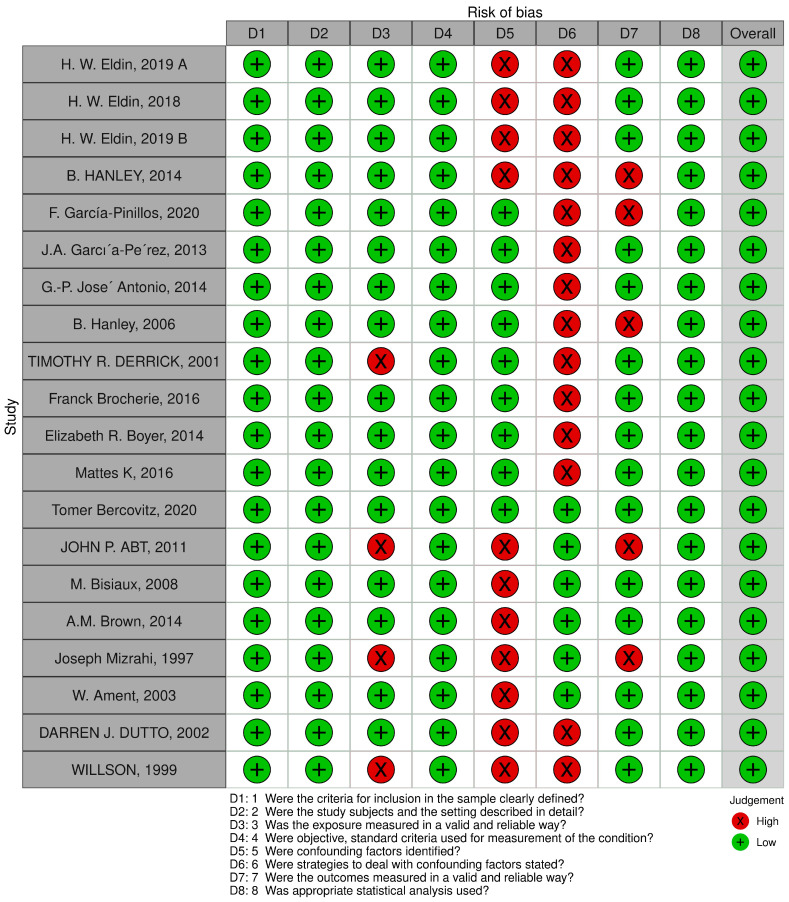
Traffic-Light Plot of Risk of Bias. “+” sign referred to low risk of bias, while “x” sign referred to high risk of bias.

**Figure 3:**
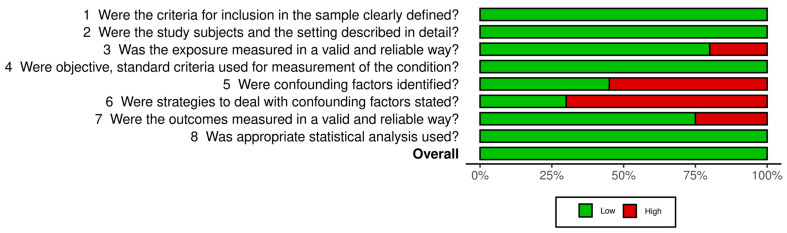
Summary Plot of Risk of Bias.

The quality assessment revealed that all the included studies [2,21,22,43,50,51,52,53,54,55,57,58,59,60,61,62,63,64,65] showed a low risk of bias. The risk of bias was presented in the traffic-light plot (Figure 2) and summary plot (Figure 3).

### 3.3. Systematic Literature Review

#### 3.3.1. Fatiguing Protocol

The fatigue protocol differed in all studies (80%) from included studies [2,21,22,43,54,55,56,57,58,59,60,61,62,63,64,65], and a localized muscle fatigue protocol was only performed in four studies (20%) from included studies [50,51,52,53]. The remaining studies differed in the fatigue protocol applied for running (treadmill, overground) and the running distances examined.

#### 3.3.2. Changes in Running Gait or Stride Characteristics with Fatigue

The studies examined a range of spatiotemporal measurements, categorized into specific items: plantar pressure, kinematic variables (e.g., contact time, step length, step frequency, flight time), kinetic variables, and accelerometer load. Table 2 provides an overview of the spatiotemporal results and the respective periods of change observed in these studies.

#### 3.3.3. Plantar Pressure

Various studies have demonstrated different findings regarding plantar pressure changes associated with fatigue. Some studies reported a reduction in plantar pressure (35%) [2,21,22,50,51,52,53] or an increase (5%) [64] with fatigue or muscular fatigue. On the other hand, certain studies found no significant change in plantar pressures (5%) [59], while others either did not measure or report plantar pressure (55%) [43,54,55,56,57,58,60,61,62,63,65].

#### 3.3.4. Kinematic Variable

The included studies evaluated a range of spatiotemporal measurements, which were grouped into various categories for analysis. These categories include:

- Contact Time

Change in contact time due to fatigue: 40% of the included studies reported a change [2,21,22,55,57,58,62,65], and 60% did not report contact time [43,50,51,52,53,54,56,59,60,61,63,64].

- Step Length

Some studies found a decrease in step length with fatigue and after muscular fatigue (35%) from included studies [21,22,50,51,52,53,65] or observed an increase (25%) from included studies [43,55,57,58,64]. Others report no significant change in step length (5%) from included studies [2] or did not measure or report step length (35%) from included studies [54,56,59,60,61,62,63].

- Stride Frequency

Some studies found an increase in stride frequency with fatigue and after muscular fatigue (50%) from included studies [21,22,43,50,51,52,53,62,64,65] or observed a decrease in stride frequency (15%) from included studies [55,57,58]. Others report no significant change in stride frequency (5%) from included studies [2] or did not measure or report stride frequency (30%) [54,56,59,60,61,63].

- Flight Time

Some studies found an increase in flight time with fatigue (15%) from included studies [57,58,65] or did not measure or report it (85%) from included studies [2,21,22,43,50,51,52,53,54,55,56,59,60,61,62,63,64].

#### 3.3.5. Kinetic Variable

Some studies found an increase in the kinetic variable with fatigue and after muscular fatigue (20%) from included studies [43,52,57,58], while others did not measure or report it (80%) from included studies [2,21,22,50,51,53,54,55,56,59,60,61,62,63,64,65].

#### 3.3.6. Accelerometer Load

Some studies found an increase in accelerometer load with fatigue (25%) from included studies [43,55,62,63,65], while others reported no significant change in accelerometer load with fatigue (10%) from included studies [56,60] or did not measure or report it (65%) from included studies [2,21,22,50,51,52,53,55,57,58,59,60,64].

#### 3.3.7. Meta-Analysis

The literature search retrieved 6626 records. We removed 2000 duplications, leaving 4626 studies for titles and abstract screening. After title and abstract screening, 201 articles were identified for full-text screening. From this, 20 studies were included in our qualitative data synthesis, and only three studies [50,51,52] (15%) from the included studies were included in our meta-analysis (Figure 1).

**Stride Frequency**

1. Right Side

**Figure 4:**
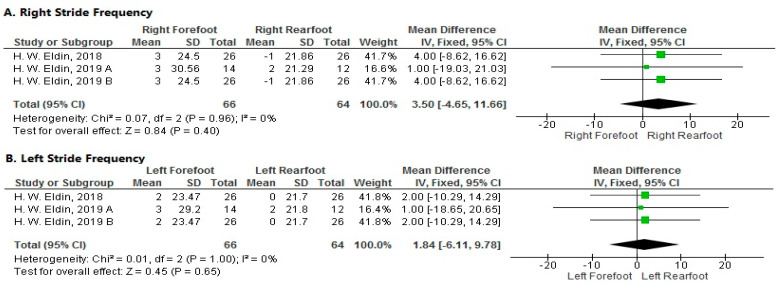
Forest plot of the studies investigating the effects of local muscle fatigue on stride frequency. CI, confidence interval; IV, instrumental variable; SD, standard deviation [50,51,52]. The green box in the middle of each horizontal line indicates a single study’s point estimate of the effect, and the size of the box is proportional to the study’s weight in relation to the pooled estimate. The black diamond indicates the meta-analysis’s overall effect estimate, while the diameter of the diamond is the 95% CI around the pooled effect point estimate.

The pooled analysis showed a non-significant increase in stride frequency of the right forefoot more than the right rearfoot after local muscle fatigue at a velocity of 15 km/h (MD = 3.5; 95% CI [−4.65–11.66]; *p* = 0.40). The pooled results were homogeneous (*p* = 0.96; I^2^ = 0%) (Figure 4A).

2. Left Side

The forest plot showed a non-significant increase in stride frequency of the left forefoot more than the left rearfoot after local muscle fatigue at a velocity of 15 km/h (MD = 1.84; 95% CI [−6.11–9.78]; *p* = 0.65). The pooled results were homogeneous (*p* = 1; I^2^ = 0%) (Figure 4B).

**Stride Length**

1. Right Side

**Figure 5:**
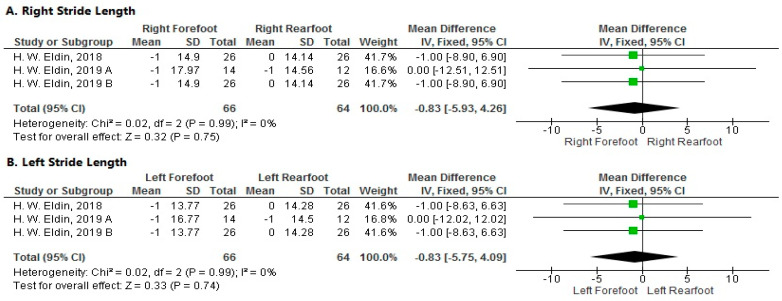
Forest plot of the studies investigating the effects of local muscle fatigue on stride length. CI, confidence interval; IV, instrumental variable; SD, standard deviation [50,51,52].

The pooled analysis showed a non-significant decrease in stride length of the right forefoot more than the right rearfoot after local muscle fatigue at a velocity of 15 km/h (MD = −0.83; 95% CI [−5.93–4.26]; *p* = 0.75). The pooled results were homogeneous (*p* = 0.99; I^2^ = 0%) (Figure 5A).

2. Left Side

The forest plot showed a non-significant decrease in stride length of the left forefoot more than the left rearfoot after local muscle fatigue at a velocity of 15 km/h (MD = −0.83; 95% CI [−5.75–4.09]; *p* = 0.74). The pooled results were homogeneous (*p* = 0.99; I^2^ = 0%) (Figure 5B).

**Pressure Maximum under the Heel**

1. Right Side

**Figure 6:**
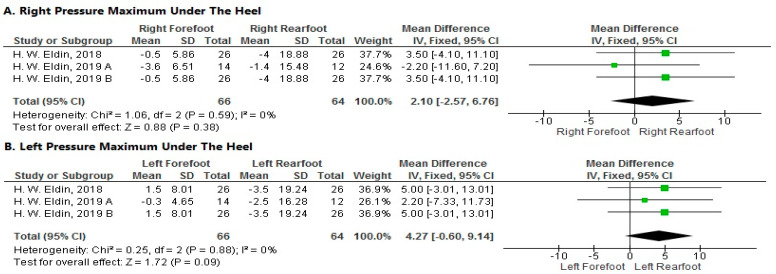
Forest plot of the studies investigating the effects of local muscle fatigue on pressure maximum under the heel. CI, confidence interval; IV, instrumental variable; SD, standard deviation [50,51,52]. The green box in the middle of each horizontal line indicates a single study’s point estimate of the effect, and the size of the box is proportional to the study’s weight in relation to the pooled estimate. The black diamond indicates the meta-analysis’s overall effect estimate, while the diameter of the diamond is the 95% CI around the pooled effect point estimate.

The pooled analysis showed a non-significant decrease of maximum pressure under the right rearfoot heel more than the right forefoot after local muscle fatigue at a velocity of 15 km/h (MD = 2.1; 95% CI [−2.57–6.76]; *p* = 0.38). The pooled results were homogeneous (*p* = 0.59; I^2^ = 0%) (Figure 6A).

2. Left Side

The forest plot showed a non-significant decrease in pressure maximum under the heel of the left rearfoot more than the left forefoot after local muscle fatigue at a velocity of 15 km/h (MD = 4.27; 95% CI [−0.6–9.14]; *p* = 0.09). The pooled results were homogeneous (*p* = 0.88; I^2^ = 0%) (Figure 6B).

**Pressure Maximum under the Metatarsus.**

1. Right Side

**Figure 7:**
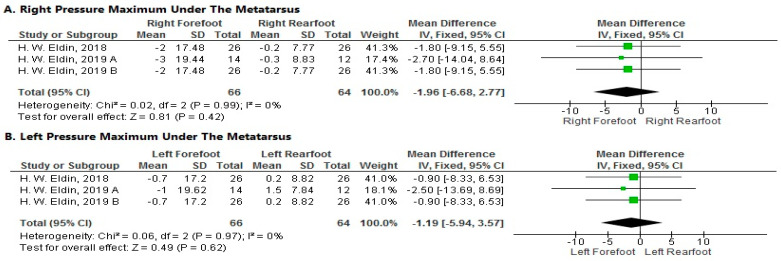
Forest plot of the studies investigating the effects of local muscle fatigue on pressure maximum under the metatarsus. CI, confidence interval; IV, instrumental variable; SD, standard deviation [50,51,52]. The green box in the middle of each horizontal line indicates a single study’s point estimate of the effect, and the size of the box is proportional to the study’s weight in relation to the pooled estimate. The black diamond indicates the meta-analysis’s overall effect estimate, while the diameter of the diamond is the 95% CI around the pooled effect point estimate.

The pooled analysis showed a non-significant decrease in pressure maximum under the metatarsus of the right forefoot more than the right rearfoot after local muscle fatigue at a velocity of 15 km/h (MD = −1.96; 95% CI [−6.68–2.77]; *p* = 0.48). The pooled results were homogeneous (*p* = 0.99; I^2^ = 0%) (Figure 7A).

2. Left Side

The forest plot showed a non-significant decrease in pressure maximum under the metatarsus of the left forefoot more than the left rearfoot after local muscle fatigue at a velocity of 15 km/h (MD = −1.19; 95% CI [−5.94–3.57]; *p* = 0.62). The pooled results were homogeneous (*p* = 0.97; I^2^ = 0%) (Figure 7B).

**Pressure Maximum under the Forefoot**

1. Right Side

**Figure 8:**
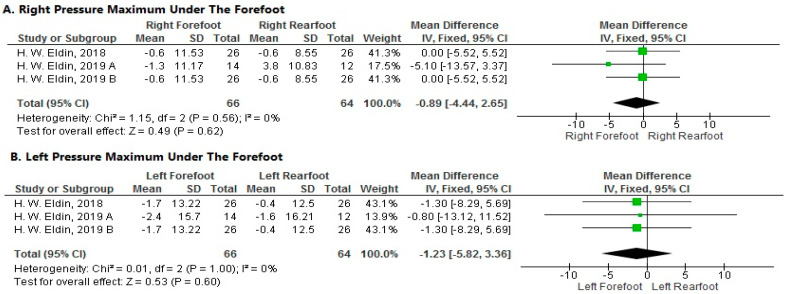
Forest plot of the studies investigating the effects of local muscle fatigue on pressure maximum under the forefoot. CI, confidence interval; IV, instrumental variable; SD, standard deviation [50,51,52]. The green box in the middle of each horizontal line indicates a single study’s point estimate of the effect, and the size of the box is proportional to the study’s weight in relation to the pooled estimate. The black diamond indicates the meta-analysis’s overall effect estimate, while the diameter of the diamond is the 95% CI around the pooled effect point estimate.

The pooled analysis showed a non-significant decrease in maximum pressure under the right rearfoot’s forefoot more than the right forefoot after local muscle fatigue at a velocity of 15 km/h (MD = −0.89; 95% CI [−4.44–2.65]; *p* = 0.62). The pooled results were homogeneous (*p* = 0.56; I^2^ = 0%) (Figure 8A).

2. Left Side

The forest plot showed a non-significant decrease in pressure maximum under the forefoot of the left rearfoot more than the left forefoot after local muscle fatigue at a velocity of 15 km/h (MD = −1.23; 95% CI [−5.82–3.36]; *p* = 0.60). The pooled results were homogeneous (*p* = 1; I^2^ = 0%) (Figure 8B).

## 4. Discussion

In this review and meta-analysis, we investigated the impact of muscle fatigue on kinematics and plantar pressure distribution during running, revealing varying results due to different fatigue protocols by conducting an extensive literature search and retrieving a total of 6626 records. After removing 2000 duplications, we were left with 4626 studies for titles and abstract screening. Subsequently, 201 articles were selected for full-text screening. Among these, 20 studies were included in our qualitative data synthesis [2,21,22,43,50,51,52,53,54,55,56,57,58,59,60,61,62,63,64,65]. However, only three studies were eligible for our meta-analysis [50,51,52]. The primary objective of this study was to evaluate how both fatigue during running and local muscle fatigue impact kinematics and plantar pressure distribution during running. We discovered that the plantar pressure distribution under the foot varies based on the fatigue protocols applied. Moreover, the results indicated differing outcomes when employing fatigue procedures involving running or jumping compared to standard muscular fatigue protocols, which produced consistent results for the ankle joint muscles.

Our results showed a non-significant increase in stride frequency of the right and left forefoot as well as the right and left rearfoot after local muscle fatigue at a velocity of 15 km/h (MD = 3.5; 95% CI [−4.65–11.66]; *p* = 0.40). Also, Alfuth et al. [25] found no changes in stride frequency. García-Pérez et al. [2] demonstrated that runners effectively mitigated fatigue effects by adjusting different components of their overall gait pattern and moderating their running speed. The study revealed that runners reduced their stride frequency similarly on both treadmill and overground surfaces (0.89% in S1 and 2.78% in S2). The experiments were conducted at two speeds: S1 = 3.33 m/s and S2 = 4.00 m/s, both before and after a fatigue protocol involving a 30-min run at 85% of their maximal aerobic speed. Gerlach et al. [15] found that stride frequency decreased significantly before and after fatigue.

The present results showed a non-significant decrease in stride length of the right and left forefoot as well as the right and left rearfoot after local muscle fatigue at a velocity of 15 km/h (MD = −0.83; 95% CI [−5.93–4.26]; *p* = 0.75). These results are different from those of Derrick et al. [43]. They found changes in running stride length after fatigue.

The current findings indicated a non-significant reduction in maximum pressure observed in both the right and left forefoot, as well as the right and left rearfoot, following local muscle fatigue; this aligns with the study by Olivier et al. [24], which reported increased forefoot pressure and decreased toe pressure. Similarly, García-Pérez et al. [2] observed alterations in plantar pressure distribution. Additionally, Willems et al. [23] documented significant changes in peak force (12.4%), mean force (11.9%), and impulse (17.6%).

Numerous publications have reached a consensus regarding the fatigue-induced increase in forefoot loading. Several studies have consistently demonstrated elevations in peak pressures and impulses beneath the metatarsals following fatigue. For instance, Alfuth et al. [25] observed a significant increase, up to 14%, under the central and lateral metatarsal heads during barefoot walking. Similarly, studies by Willems et al. [23] and Weist et al. [26] reported greater peak pressures due to fatigue across the whole arch, particularly under the medial arch, leading to an increased relative load on the medial arch. García-Pérez et al. [2] also contributed to this understanding. Moreover, Bisiaux et al. [21] found a significant rise in pressure peak and relative impulse under the forefoot 30 min after running. Further supporting this, Weist et al. [26] observed increased maximal force (5%, *p* < 0.01), peak pressure (12%, *p* < 0.001), and impulse (9%, *p* < 0.01) under the second and third metatarsal heads and the medial midfoot towards the end of a fatiguing run. They also highlighted a significant increase in pressure under the medial midfoot.

Inconsistencies regarding the effects of fatigue on different foot regions have been noted, particularly concerning the heel, midfoot, and toes [23]. Some studies did not find significant changes in peak pressures due to running [25]. It is worth noting that fatigue may impact the biomechanical pattern of running differently on a treadmill compared to overground, given the distinct muscle activity patterns and specific neuromuscular control mechanisms for each surface [66]. Interestingly, several studies reported reduced peak pressures across various foot zones, with significant differences observed in the whole foot and lateral heel and a slight trend in the hallux [2]. This reduction could be attributed to runners adapting their running kinematics to sustain fatigued performance [30].

Additionally, certain studies indicated a decrease in peak pressures under the toes and an increase in the metatarsal area, suggesting that local fatigue of the toe flexors might drive this pressure shift [23,25,67]. Despite no metatarsal overload, García-Pérez et al. [2] observed a significant reduction in peak pressure under the lateral heel and hallux. This alteration in the “roll-over process” due to fatigue could explain the observed change in plantar pressure distribution [2]. Consistent with these findings, previous studies have also reported decreased peak pressures under the hallux and heel due to fatigue [22,67]. This behavior, coupled with the observed load increase under the medial heel by Willems et al. [23], might indicate greater foot pronation resulting from local muscular fatigue, particularly fatigue of the tibialis posterior, known to exhibit fatigue during running motion [22,23].

The noted increase in foot pronation resulting from fatigue could potentially elevate the risk of injuries, a common occurrence among runners. During the forefoot push-off phase, there was a notable shift towards a more lateral pressure distribution. However, inconsistencies were observed regarding the effects of fatigue on the heel, midfoot, and toes [23]. Nevertheless, there is a consensus regarding the fatigue-induced escalation of forefoot loading. Multiple studies have demonstrated rises in peak pressures and impulses under the metatarsals [21,22,26,67]. A study by [67] described a reduction in pressure under the toes post-marathon race, while another study by [26] found no differences.

Weist et al. [26] reported a notable rise in pressure under the medial midfoot. Conversely, Alfuth et al. [21] observed decreased peak pressure and relative impulses under the midfoot after 30 min of intensive running. Moreover, Weist et al. [26] found increased impulse underneath the medial heel, contrasting with studies such as those by [6,22], which indicated a decrease in rearfoot loading following a fatiguing run. Additionally, Bisiaux et al. [21] found significant reductions in peak pressures and relative impulses under the heel after 30 min of intensive running.

The tibialis posterior muscle is crucial for controlling rearfoot eversion and providing dynamic support across the midfoot and forefoot during the stance phase of gait [68,69]. Noteworthy changes were observed, including significant increases in peak force (12.4%), mean force (11.9%), and impulse (17.6%), coupled with a general increase in metatarsal loading [23]. Fatigue in runners leads to a higher relative load on the medial arch and a decrease in peak pressure under the lateral heel and hallux [2]. However, forces beneath the lateral heel did not show significant differences [2]. Within the midfoot region, there was no Table 11.7% increase in impulse (*p* = 0.021), while peak force and mean force significantly decreased beneath toes two to five by 8.1% and 7.2%, respectively. Forces under the hallux did not demonstrate significant differences [56,65]. Weist et al. [26] showcased a significant increase in impulse under the medial heel, although peak and mean forces did not experience significant changes. They also observed a substantial increase in loading under the midfoot and medial heel. Willems et al. [23] reported heightened loading under the medial midfoot, impulse under the medial heel, and peak and mean force after a 20 km race.

Following local muscle fatigue in the meta-analysis, our findings indicate an insignificant reduction in maximum pressure. Other studies also reported diminished pressure values [2,21,23]. This result supports the hypothesis proposing a potential strategy to reduce the risk of fatigue injuries [10]. It is attributed to several factors, including decreased vertical movement of the body’s center of gravity, reduced muscle stiffness, enhanced shock absorption due to increased knee joint flexion [36], and a flattened foot arch caused by dorsiflexion fatigue [17]. These factors collectively contribute to a reduction in landing pressure.

Moreover, as the running speed remained constant with no alterations in stride frequency or length, the load on the forefoot decreased, further diminishing muscular fatigue during running. Furthermore, it is essential to consider the duration for which this compensatory mechanism remains effective without impacting running speed and its applicability within the limited time frame of our treadmill-based studies, which typically last only 60 s. Elevated pressure values beneath the heel point cause increased muscle stiffness and reduced compliance during foot strikes, leading to a greater load on the heel of the more powerful leg. However, it is important to note that these findings exhibited only a small effect size and lost statistical significance after adjusting for multiple comparisons (P adjustment). The fatigue protocol revealed that the dorsiflexor muscles displayed a notably higher level of fatigue when compared to the plantar flexors, thereby exerting an impact on the muscular control of the ankle. One reason for this emerging muscular imbalance can be attributed to the greater strength of the plantar flexors, owing to their larger muscle mass and distinct roles in running and walking. Running places more stress on the plantar flexors, enhancing their fatigue resistance over time. EMG analyses reveal that fatigue alters the neuromuscular relationship in movement control, impacting rolling behavior and plantar pressure distribution in foot movement [41]. Surprisingly, weaker dorsiflexors following fatigue did not correlate with a higher plantar pressure load but resulted in reduced plantar pressure under the heel. This decrease in heel pressure might be attributed to the weakened support function of the dorsiflexors when flattening the foot and attaching it to the ground, potentially coupled with increased flexion in the knee joint [70]. These alterations in load distribution affect various aspects of the foot, ankle, knee joint, and stabilizing muscles. Notably, there is a stress reduction, at least for the foot, supporting adopting a protective strategy to mitigate fatigue-related injuries.

Nonetheless, it is important to consider the treadmill load protocol, which involved short running durations of approximately 1 min per running speed and required subjects to maintain increased attention and focus. This approach led to a reduction in stress, which was more pronounced at the examined running speeds. Interestingly, even after local muscle fatigue at the same running speed, no significant changes were observed in stride frequency or stride length, aligning with the findings by Alfuth and Rosenbaum [25], who also reported no alterations in determined step frequency [25].

Additionally, Willems et al. [23] observed that following a 20 km race, the first contact time of the metatarsal occurred earlier, and the total foot contact time was significantly longer. In a separate study, Weist et al. [26] noted only minimal effects on contact area and contact time, alongside a decrease in step time, as reported [22]. Some studies also indicated an increase in flight time with fatigue [57,58,65], which aligns with the findings by Mizrahi et al. [41].

Previous research consistently indicates an increase in the kinetic variable time with fatigue and after experiencing muscular fatigue [43,51,57,58]. These findings align with those of [15,17], illustrating well-documented kinetic changes following fatigue in both healthy and injured runners. However, two additional studies [71,72] suggest differences in both kinematics and kinetics between treadmill and overground locomotion. Another study by Brown et al. [61] found no interaction between fatigue and limb dominance when examining joint kinematics or kinetics. Consequently, fatigue may accentuate the kinematic and kinetic differences between limbs. Moreover, muscular fatigue has been linked to delayed muscle activation and subsequent kinematic and kinetic alterations in the running stride [43].

Based on this research, using different fatigue protocols leads to different results. Thus, we cannot compare the results of different studies except in the case of a standard muscle fatigue protocol for the knee and ankle joint through which experiments are conducted; thus, comparisons can be made between those results.

### Limitations

The treadmill studies are subjected to the disadvantage of unfamiliar conditions affecting the running movement, but the laboratory situation simplifies the control of disturbances and data collection. Another limitation is the plantar pressure measurement, which only included the vertical force registered with lower measurement accuracy than force plates but averaged several running cycles over a duration of 30 s. Using the manufacturer’s software, a reduction to three-foot zones without separating the toes of the medial and lateral foot is restricted to comparability with other studies. The fundamental relationship between the movement technique of the lower extremities and plantar pressure distribution is still not fully understood or has yet to be established.

The observed trend of forefoot strikes during barefoot running on the treadmill suggests that some individuals classified as forefoot runners may not consistently maintain this foot strike pattern when wearing running shoes; this highlights the importance of considering footwear in foot strike pattern analysis. Additionally, the exclusive focus on male participants in the conducted experiments underlines the necessity for further research involving groups of women. Comparative studies between genders can provide a more comprehensive understanding of potential variations in foot strike patterns based on gender. Regarding research methodology, this study relied solely on cross-sectional studies. However, incorporating diverse study designs, such as cohort studies, case-control studies, cross-over studies, and randomized controlled trials, can provide a more robust and comprehensive analysis of foot strike patterns and their implications. Diversifying the study designs can enhance the reliability and generalizability of the findings in this area of research.

## 5. Conclusions

Our results suggest that experiments should use a standard muscle fatigue protocol and a standard running fatigue protocol to have reliable results when comparing plantar pressure distribution and kinetic. A deficiency in the procedure for assessing local muscle fatigue of the knee joint muscles was found. Therefore, it is recommended to use a standard protocol of local muscle fatigue for knee and ankle joints to compare results from different studies. In addition to running, strength training of the foot muscles with a specific focus on the dorsiflexor muscles should be done to prevent running-related injuries. One of the future targets should be applying these experiments while wearing shoes, which is the main running method. The foot strike patterns should be varied for injury prevention to relieve the foot area under the heel. To get a better analysis of the plantar pressure values, it is recommended to check the kinematic parameters of the angles of the knee joint, ankle, and sole for a better understanding of the relationship between the plantar pressure distribution and the movement of the lower limbs. 

## Figures and Tables

**Table 1:** Eligibility Criteria.

Criterion	Description
Type of participant	Healthy adult runners (all competitive levels, all sexes).
Type of comparison	Effect analysis (fatigue by jumping, fatigue by running, and local muscle fatigue) or regression analysis (running biomechanics).
Type of outcome measure	Plantar pressure distribution, running biomechanics (kinematics, kinetics, and EMG outcomes), temporal-spatial data
Type of Study	cross-sectional studies will be included
Publication status	Peer-reviewed journal publication
Publication date	The included studies were not restricted to a specific date of publication.
Language of publication	English or German language

**Table 2:** Summary of Including Studies (N = 20).

Study ID	Country	Study Design	Sample Size	Inclusion Criteria	Aim	Outcomes		Conclusion
						Primary Outcome	Secondary Outcome	
Garcı’a-Pe´rez et al. [2].	Spain	Cross-Sectional Study	27 (10 females, 17 males)	Fatigue During Running	To examine the impact of treadmill versus overground conditions on plantar pressures before and after experiencing fatigue.	On both types of surfaces, fatigue (S2) resulted in a decrease in stride frequency (2.78%) and a reduction in plantar pressure (PP) on the lateral heel and hallux (15.96% and 16.35%, respectively). Fatigue (S1) also increased the relative load on the medial arch (9.53%).	No notable interaction was observed between the two factors under investigation, namely surface type and fatigue.	The influence of surface type, irrespective of fatigue levels, should be considered when interpreting findings from studies incorporating treadmill use in their experimental designs or recommending physical exercise on a treadmill.
Bisiaux et al. [21].	France	Cross-Sectional Study	11 males	30-Min After Intensive Running	To evaluate alterations in plantar pressure caused by fatigue.	Significant reductions in pressure peaks and relative impulses were noted under the heel and midfoot, accompanied by a noteworthy increase in pressure peaks and relative impulses under the forefoot 30 min post-run.	Following a 30-min rest, there was a significant alteration in loading at both the heel and forefoot compared to pre-test conditions, while variability, step length, and frequency remained consistent.	The study illustrates short- and long-term variations in plantar pressure caused by fatigue resulting from an intensive 30-min run; this contrasts with prior studies that indicated minimal changes in ground reaction force.
Willson et al. [22].	USA	Cross-Sectional Study	19 (11 females, eight males)	Fatigue During Running	To pinpoint alterations in foot loading patterns associated with fatigue during running	The reduction in step time was accompanied by significantly lower plantar pressure values under the heel.	A discernible shift towards heightened medial forefoot loading was detected when subjects ran under fatigued conditions (α < 0.05).	The subjects exhibited an alteration in running technique and plantar surface loading characteristics in response to fatigue, characterized by an augmented cadence, reduced heel loading, and heightened medial forefoot loading.
Derrick et al. [43].	USA	Cross-Sectional Study	Ten males	Fatigue During Running	To investigate the kinematic adaptations runners make during an exhaustive run and their impact on shock absorption and attenuation.	The knee exhibited a notable increase in flexion at heel impact (initial: 164.9 ± 2.3°; final: 160.5 ± 2.9°; *p* < 0.05).	The rearfoot angle displayed increased inversion at impact (initial: 12.2 ± 1.6°; final: 13.6 ± 1.9°; *p* < 0.05).	The heightened peak impact accelerations at the leg were not regarded as an augmented injury risk due to the reduced effective mass. However, the modified kinematics may have led to heightened metabolic costs during the latter stages of the exhaustive run.
Hazzaa et al. [50].	Germany	Cross-Sectional Study	52 males	Local Muscle Fatigue	The study explored how the foot strike pattern and local muscle fatigue of the plantar flexors and dorsiflexors impact plantar pressure distribution during barefoot treadmill running at three distinct speeds.	Plantar pressure distribution across the foot varied based on the foot strike pattern and local muscle fatigue.	The maximum pressure experienced in the exposed regions of the foot decreased after fatigue.	Injury prevention strategies should encompass variations in foot strike patterns to alleviate pressure on the heel and forefoot areas; this is particularly relevant for forefoot strikers.
Hazzaa et al. [51].	Germany	Cross-Sectional Study	26 males	Local Muscle Fatigue	The study examined how foot strike patterns and local muscle fatigue of the plantar and dorsiflexors influence plantar pressure distribution and selected kinematic characteristics during barefoot treadmill running at three different speeds.	Following fatigue, the maximum pressure experienced in the exposed foot regions decreased, specifically under the forefoot in the case of a forefoot strike and under the heel for a rearfoot strike.	The two groups of runners exhibited variations in foot angle at foot strike, with forefoot runners displaying higher values.	The increased foot angle observed in forefoot runners enhances shock absorption, potentially reducing the risk of injury.
Hazzaa et al. [52].	Germany	Cross-Sectional Study	52 males	Local Muscle Fatigue	The aim was to evaluate plantar pressure deviations attributed to fatigue and the specific foot strike pattern.	Both groups exhibited significant disparities in plantar pressure distribution at baseline and after experiencing fatigue.	In both running groups, as running speed increased, there was an associated rise in stride length, stride frequency, and maximum plantar pressure under the midfoot and forefoot.	To prevent injuries, it is recommended to strengthen the plantar muscles of forefoot strikers through targeted strength training.
Mattes et al. [53].	Germany	Cross-Sectional Study	30 males	Local Muscle Fatigue	Considering leg strength asymmetries, the study investigated how a standardized fatigue protocol affects plantar pressure distribution in rearfoot strike runners.	The observed reductions in pressure values suggest a potential protective strategy to mitigate injuries during muscle fatigue.	Leg asymmetry was present, and the fatigue protocol had a more pronounced effect on dorsiflexor performance, likely because plantar flexors, benefiting from their larger muscle mass, exhibited higher fatigue resistance.	The observed decrease in pressure values could suggest a potential protective mechanism aimed at mitigating injuries during muscle fatigue.
Bercovitz et al. [64].	Israel	Cross-Sectional Study	Nine males	Fatigue During Running	The objective was to evaluate changes in plantar pressure and center of pressure (COP) trajectory after a 30-min run at sub-maximal speed in experienced long-distance runners.	Substantial alterations in the plantar pressure map were identified post-run, characterized by heightened impulses in the first metatarsal head (9.92%, *p* < 0.001) and hallux areas (16.19%, *p* < 0.001), along with reduced impulses in the fourth and fifth metatarsal heads (4.95%, *p* < 0.05).	The center of pressure (COP) curve demonstrated a significant medial shift (*p* < 0.01). The plantar pressure map and COP trajectory changed after a 30-min exhaustive run.	These alterations might signify heightened stress on joints and tissues when individuals are fatigued, potentially increasing the risk of overload injuries.
Boyer et al. [59].	USA	Cross-Sectional Study	30 (15 females, 15 males)	Fatigue During Running	The study aimed to assess alterations in the arch mechanics of healthy runners before and after a 45-min run at a comfortable pace. The hypothesis was that there would be increased arch deformation and reduced arch stiffness following the run.	The study found no statistically significant differences in the mean (95% confidence interval) values for navicular displacement (5.6 mm [4.7–6.4 mm]), arch lengthening (3.2 mm [2.6–3.9 mm]), change in arch height index (0.015 [0.012–0.018]), or arch rigidity index (0.95 [0.94–0.96]) following the 45-min run.	The multivariate analyses of variance did not yield statistically significant differences, with all *p*-values (*p*) being greater than or equal to 0.065.	Given the absence of statistically significant changes in arch deformation or rigidity, it can be concluded that the structures of a healthy and intact medial longitudinal arch can adapt to cyclical loading, such as enduring a 45-min run, without undergoing compromise.
Abt et al. [60].	USA	Cross-Sectional Study	12 (six females, six males)	Fatigue During Running	The aim was to investigate whether an exhaustive running about at physiologically determined high-intensity levels causes changes in running kinematics, impacts accelerations, and alters shock attenuating capabilities.	No significant differences were observed for this study’s kinematic or acceleration variables.	While the results of this study did not align with the initial hypotheses, it is essential to note that the impact of running fatigue on kinematics and accelerations remains inconclusive. Further research and exploration are warranted to understand this relationship better.	Future research should investigate fatigue-induced alterations in running kinematics and accelerations to pinpoint the threshold at which these changes manifest.
Ament et al. [54].	Netherland	Cross-Sectional Study	13 males	Fatigue Running on Treadmill to Exhaustion	The objective was to ascertain the speed and accuracy with which individuals could use visual cues to adapt to the impact position of their feet.	This involved plotting the average adjustment of foot impact position for a single subject. The plot displayed the relative longitudinal foot impact position on the treadmill plate (measured in centimeters) against step number, averaging all the relevant target-line switching cycles throughout a full test run.		The findings revealed prolonged multi-step motor adjustment processes during treadmill running, highlighting the sensitivity of foot placement to the task’s duration as individuals neared exhaustion.
Brocherie et al. [55].	SWITZERLAND	Cross-Sectional Study	13 males	Fatigue Running on a Treadmill	Strong evidence indicates greater performance decrements during repeated sprinting in hypoxic (low oxygen) conditions than in normal (normoxic) conditions.	The study found that the sprint decrement score, which indicates the decline in sprint performance, was comparable across different conditions. The pooled values for the sprint decrement score showed no significant difference, with an average of 11.4 ± 7.9% (*p* = 0.49).	The study found that fatigue-induced changes in standard shoes (SH) differed from those observed in minimalist shoes (SL) and maximalist shoes (MH). Specifically, fatigue in SH resulted in more pronounced alterations in repeated sprint ability, including kinetics, kinematics, and spring-mass characteristics, compared to SL and MH. These alterations were characterized by a reduced ability to effectively apply forward-oriented ground reaction force, maintain vertical stiffness, and sustain stride frequency during repeated sprints in SH. In contrast, SL and MH showed either no or minimal changes in these aspects following fatigue.	In the study, it was observed that impairments in repeated sprint ability and the associated kinetics, kinematics, and spring-mass characteristics were more pronounced in SH (standard shoes) compared to SL (minimalist shoes) and MH (maximalist shoes). The differences were notable, particularly in SH, where fatigue during repeated sprints reduced the ability to effectively apply forward-oriented ground reaction force, maintain vertical stiffness, and sustain stride frequency. On the other hand, SL and MH showed either no or minimal differences in these aspects.
Brown et al. [61].	USA	Cross-Sectional Study	20 females	Fatigue During Running	To establish whether lower extremity limb dominance influences overground running mechanics.	There were no significant differences between the kinematic or kinetic patterns of the dominant and non-dominant lower extremities during fresh-state overground running.	Fatigue was not shown to interact with limb dominance.	Limb dominance did not affect kinematic or kinetic side-to-side differences. Therefore, physical therapists can continue to use a resolution of lower extremity symmetry as a goal of therapy without having to account for limb dominance.
Dutto et al. [62].	USA	Cross-Sectional Study	15 (4 females, 11 males)	Fatigue Running on Treadmill to Exhaustion	The objective was to investigate if leg stiffness characteristics undergo alterations during a treadmill run to voluntary exhaustion.	The group analysis demonstrated a significant decrease (*p* = 0.01) in both vertical (from 23.9 to 23.1 kn·m^−1^) and leg (from 9.3 to 9.0 kn·m^−1^) stiffness over the course of the run.	Ten of the 15 runners experienced statistically significant changes in their stride rate.	The observed changes in stride rate are likely a result of alterations in the stiffness characteristics of the leg during a run to fatigue.
García-Pe´rez, et al. [56].	Spain	Cross-Sectional Study	20 (9 females, 11 males)	Fatigue Running on a Treadmill	To examine the impact acceleration and shock attenuation before and after fatigue during treadmill running.	Treadmill running reduced both head and tibial peak impact accelerations and impact rates, yet no significant distinction was noted in shock attenuation between the two surfaces.	A significant interaction was observed between the surface (treadmill and overground) and fatigue state (pre-fatigue and post-fatigue).	The impact of treadmill running and its interaction with other variables should be considered when interpreting the outcomes of studies utilizing treadmill-based experimental protocols and designing exercise prescriptions.
García-Pinillos et al. [65].	Chile	Cross-Sectional Study	22 males	Fatigue During Running	To investigate how a running protocol inducing fatigue affects spatiotemporal gait parameters, step variability, vertical (Kvert), and leg stiffness (Kleg) during treadmill running.	Pairwise comparisons, specifically between non-fatigued and fatigued conditions, revealed significant alterations in temporal parameters (i.e., CT and FT) with *p*-values of 0.001 and less than 0.001, respectively.	In the fatigued condition, Kleg demonstrated a significant reduction (*p* < 0.001), whereas Kvert remained unchanged (*p* = 0.602).	The findings suggest that a 60-min trial run induces adaptations in spatiotemporal gait characteristics and stiffness due to fatigue among trained endurance runners.
Hanley et al. [57].	United Kingdom	Cross-Sectional Study	13 males	Fatigue During Running	The objective was to quantify the impact of fatigue on gait parameters during running.	Significant variations were noted in maximum force, impulse, and contact time (*p* < 0.01).	Dependent t-tests revealed a notable difference in the knee angle at take-off (*p* < 0.01). The kinetics and temporal aspects alterations were noticeable as early as the 3000 m mark.	Athletes are encouraged to maintain a consistent race pace to mitigate the impact of fatigue.
Hanley et al. [58].	United Kingdom	Cross-Sectional Study	15 males	Fatigue Running on treadmill	The aim was to assess alterations in gait parameters during a 10-km treadmill run.	Before reaching the halfway point, there was an increase in step length and a decrease in cadence.	In the latter stages, notable reductions were observed in impulse and maximum force. Concurrently, contact time decreased, and flight time consistently increased. However, the majority of gait variables remained relatively stable.	Running at a constant pace on a treadmill can be beneficial for maintaining a specific distance and speed while keeping a consistent technique unaffected by variables like gradient or fatigue.
Mizrahi et al. [63].	USA	Cross-Sectional Study	22 males	Fatigue During Running	The study investigated how fatigue impacts the human musculoskeletal system’s ability to absorb shock waves generated during heel strikes. Additionally, the research sought to establish correlations between changes in the electromyography (EMG) signal and other fatigue measures.	The study observed a consistent rise in acceleration signal amplitude when experiencing general fatigue.	No correlation was observed between EMG activity and fatigue in the time or frequency domains.	In conclusion, fatigue appears to diminish the ability of the human musculoskeletal system to absorb the shock acceleration resulting from heel strikes effectively.

## Data Availability

The data extraction sheet is available at the editor’s request. Data are contained within the article and Supplementary Materials.

## Supplementary Materials

The following supporting information can be downloaded at: https://www.mdpi.com/article/10.3390/sports11120241/s1, PRISMA checklist as the supplementary materials
